# The Challenge of Delivering Therapeutic Aerosols to Asthma Patients

**DOI:** 10.1155/2013/102418

**Published:** 2013-08-05

**Authors:** Federico Lavorini

**Affiliations:** Department of Experimental and Clinical Medicine, Careggi University Hospital, Largo Brambilla 3, 50134, Florence, Italy

## Abstract

The number of people with asthma continues to grow around the world, and asthma remains a poorly controlled disease despite the availability of management guidelines and highly effective medication. Patient noncompliance with therapy is a major reason for poor asthma control. Patients fail to comply with their asthma regimen for a wide variety of reasons, but incorrect use of inhaler devices is amongst the most common. The pressurised metered-dose inhaler (pMDI) is still the most frequently used device worldwide, but many patients fail to use it correctly, even after repeated tuition. Breath-actuated inhalers are easier to use than pMDIs. The rationale behind inhaler choice should be evidence based rather than empirical. When choosing an inhaler device, it is essential that it is easy to use correctly, dosing is consistent, adequate drug is deposited in both central and peripheral airways, and that drug deposition is independent of airflow. Regular checking of inhalation technique is crucial, as correct inhalation is one of the cornerstones of successful asthma management.

## 1. Introduction 

The incidence of asthma continues to rise worldwide, doubling over the last 10 years [1–4] and, consequently, asthma places a huge economic burden on healthcare resources [5]. Asthma management guidelines [1, 2] are now available in virtually every country; their aim is to achieve control of the disease with the lowest possible dose of medication prescribed [1, 2]. To this end, asthma guidelines advocate a stepwise pharmacological approach that consists in increasing (“step up”) the numbers of medications as asthma worsens, and decreasing (“step down”) medications when asthma is under control [1, 2]. Once control of asthma has been achieved and maintained for at least three months, a gradual reduction of the maintenance therapy is recommended to identify the minimum therapy required to maintain control [1, 2]. Unfortunately, the current level of asthma control falls far short of the goals for long-term asthma management [2, 6, 7], with many patients reporting day- and night-time symptoms at least once a week, and continuing to require unscheduled visits and hospitalisations [2, 6, 7]. One of the reason why asthma remains poorly controlled is that patients are deriving incomplete benefit from their inhaled medication, primarily because they are unable to use their inhalers correctly [8–11]. 

The benefits of inhaled therapy for the treatment of obstructive airway diseases, such as asthma, have been recognised for many years. In comparison with oral or parenteral formulations, minute but therapeutic doses of drug are delivered topically into the airways causing local effects within the lungs [12–14]. Unwanted systemic effects are minimised as the medication acts with maximum pulmonary specificity together with a rapid onset and duration of action [12–14]. Consequently, aerosol formulations of bronchodilators and corticosteroids are the mainstay of modern treatment for asthma at all ages [1, 2]. Aerosols are either solutions containing medications, or suspensions of solid particles in a gas, generated from devices such as pressurised metered dose inhalers (pMDIs), dry powder inhalers (DPIs) or nebulisers [12–16]. In the past decade some novel delivery systems have been developed that have high delivery efficiencies; notable among these are the soft mist inhalers (SMI). Each type of inhaler device has *pros* as well as *cons* (Table 1). Inhalers differ in their efficiency of drug delivery to the lower respiratory tract, depending on the form of the device, its internal resistance, formulation of medication, particle size, velocity of the produced aerosol plume, and ease with which patients can use the device [12–16]. Efficiency of drug delivery may also be influenced by patients' preference, which in turn affects patients' adherence with treatment and indeed long-term control of the disease [17]. There seems little point in prescribing an effective medication in an inhaler device which patients cannot use correctly. Thus, the choice of the right inhaler for the patient is just as important as choosing the most effective medication. 

In this paper, the hand-held inhalers are reviewed together with a current understanding about correct inhalation techniques for each device. A description of nebulisers, which are frequently used to deliver asthma medications [18], is also given. However, since most current nebuliser designs are bulky and inconvenient and drug administration is prolonged, they are better categorised as second-line devices for most asthma patients. Finally, we present recommendations from the *Aerosol Drug Management Improvement Team* (ADMIT) for inhaler selection, as well as an algorithm for asthma therapy adjustment [8]. 

## 2. Aerosol Device Options 

### 2.1. Pressurised Metered-Dose Inhalers

**Figure 1:**
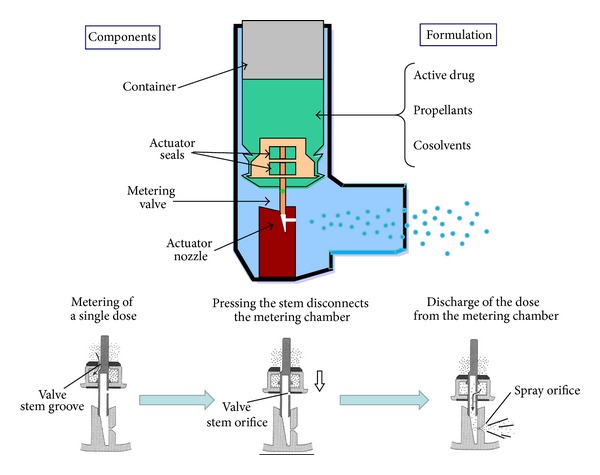
Components of a pressurised metered-dose inhaler. Lower panels illustrate the process of aerosol generation.

The development of the first commercial pMDIs was carried out by Riker Laboratories in 1955 and marketing in 1956 as the first portable, multidose delivery system for bronchodilators. Since that time, the pMDI has become the most widely prescribed inhalation device for drug delivery to the respiratory tract to treat obstructive airway diseases such as asthma and chronic obstructive pulmonary disease [18]; the total worldwide sales by all companies of pMDI products run in excess of $2 billion per year. The pMDI (Figure 1) is a portable multidose device that consists of an aluminium canister, lodged in a plastic support, containing a pressurised suspension or solution of micronized drug particles dispersed in propellants. A surfactant (usually sorbitan trioleate or lecithin) is also added to the formulation to reduce the particle agglomeration and is responsible for the characteristic taste of specific inhaler brands. The key component of the pMDI is a metering valve, which delivers an accurately known volume of propellant, containing the micronised drug at each valve actuation. Pressing the bottom of the canister into the actuator seating causes decompression of the formulation within the metering valve, resulting in an explosive generation of a heterodisperse aerosol of droplets that consist of tiny drug particles contained within a shell of propellant. The latter evaporates with time and distance, which reduces the size of the particles that use a propellant under pressure to generate a metered dose of an aerosol through an atomisation nozzle (Figure 1). The technology of pMDI has evolved steadily over the period of mid-1950s to the mid-1980s. Until recently, the pMDI used chlorofluorocarbons (CFCs) as propellants to deliver drugs; however, in accordance with the Montreal Protocol of 1987, CFC propellants are being replaced by hydrofluoroalkane (HFA) propellants that do not have ozone depleting properties [19]. Hydrofluoroalkane 134a and 227ca are propellants that contain no chlorine and have a residence in the stratosphere lower than CFCs, so they have substantially less global warming potential than do CFCs. HFA-134a albuterol has been the first HFA-driven pMDI that has received approval in both Europe and the United States. This preparation consists of albuterol suspended in HFA-134a, oleic acid, and ethanol; clinical trials have shown this preparation to be bioequivalent to CFCs albuterol in both bronchodilator efficacy and side effects [20]. At the present, in most European countries CFC-driven pMDIs have totally been replaced by HFA inhalers. The components of CFC-driven pMDIs (i.e., canister, metering valve, actuator, and propellant) are retained in HFA-driven pMDIs, but they have had a redesign. Two approaches were used in the reformulation of HFA-driven pMDIs. The first approach was to show equivalence with the CFC-driven pMDI, which helped regulatory approval, delivering salbutamol and some corticosteroid. Some HFA formulations were matched to their CFC counterparts on a microgram for microgram basis; therefore, no dosage modification was needed when switching from a CFC to an HFA formulation. The second approach involved extensive changes, particularly for corticosteroid inhalers containing beclomethasone dipropionate, and resulted in solution aerosols with extra-fine particle size distributions and high lung deposition [21, 22]. The exact dose equivalence of extra-fine HFA beclomethasone dipropionate and CFC beclomethasone dipropionate has not been established, but data from most trials have indicated a 2 : 1 dose ratio in favour of the HFA-driven pMDI [21, 22]. Patients on regular long-term treatment with a CFC pMDI could safely switch to an HFA pMDI without any deterioration in pulmonary function, loss of disease control, increased frequency of hospital admissions, or other adverse effects [19]. However, when physicians prescribe HFA formulations in place of CFC versions for the first time, they should inform their patients about differences between these products. Compared with CFC-driven pMDIs, many HFA-driven pMDIs have a lower (25.5 mN versus 95.4 mN) impact force and a higher (8°C versus −29°C) temperature [12, 14]. These properties partially overcome the “cold Freon effect” [12, 14] that has caused some patients to stop inhaling their CFC pMDIs. In addition, most HFA pMDIs have a smaller delivery orifice that may result in a more slowly delivered aerosol plume, thus facilitating inhalation and producing less mouth irritation [23]. Another difference is that many HFA-driven pMDIs contain small amount of ethanol. This affects the taste, as well as further increasing the temperature and decreasing the velocity of the aerosol. Pressurized MDIs containing fixed combination of beclomethasone dipropionate and the long-acting bronchodilator formoterol in a solution formulation with HFA-134a and ethanol with cosolvent [21, 24, 25] have been developed (Modulite technology, Chiesi, Italy). Interestingly, this formulation dispenses an aerosol that has a particularly small particle size (mass median aerodynamic diameter ~1 *μ*), a lower plume velocity of the aerosol, and not dropping temperature as much as when CFCs are used as carriers. These three factors, that is, smaller particle size, lower plume velocity, and less temperature drop, may decrease upper airway impaction and increase airway deposition of particles, particularly to the smaller airways, compared with the same dose of drug administered from a CFC pMDI [24, 25].

Pressurised MDIs have a number of advantages (Table 1): they are compact, portable, relatively inexpensive, and contain at least 200 metered doses per canister that are immediately ready for the use. Furthermore, a large fraction (approximately 40%) of the aerosol particles is in the respirable range (mass median aerodynamic diameter less than 5 *μ*), and dosing is generally highly reproducible from puff to puff [12–16]. Despite these advantages, most patients cannot use pMDIs correctly, even after repeated tuition [8–11]. This is because pMDIs require good coordination of patient inspiration and inhaler actuation to ensure correct inhalation and deposition of drug in the lungs. The correct inhalation technique when using pMDIs involves firing the pMDI while breathing in deeply and slowly, continuing to inhale after firing, and then following inhalation with a breath-holding pause to allow particles to sediment on the airways [12, 26]. The patients should also be instructed that, on the first use and after several days of disuse, the pMDI should be primed. However, patients frequently fail to continuously inhale slowly after activation of the inhaler and exhale fully before inhalation [8]. In addition, patients often activate the inhaler before inhalation or at the end of inhalation by initiating inhaler actuation while breath holding [8]. Crompton and colleagues [8, 27, 28] showed that the proportion of patients capable of using their pMDIs correctly after reading the package insert fell from 46% in 1982 to 21% in 2000, while only just over half of patients (52%) used a pMDI correctly even after receiving instruction. In a large (*n* = 4078) study, 71% of patients were found to have difficulty using pMDIs, and almost half of them had poor coordination [29]. Incorrect inhalation technique was associated with poor asthma control and with poor pMDI users having less stable asthma control than good pMDI users [29]. 

Even with correct inhalation technique, pMDIs are inefficient since no more than 20% for CFC pMDIs or 40%–50% for HFA pMDIs producing extra-fine particles [12, 14–16] of the emitted dose reaches the lungs with a high proportion of drug being deposited in the mouth and oropharynx which can cause local as well as systemic side effects due to rapid absorption [12, 14–16]. Another disadvantage of some pMDIs is the absence of built-in counters that would alert the patient to the fact that the inhaler was approaching “empty” and needed to be refilled. Although many pMDIs contain more than the labelled number of doses, drug delivery per actuation may be very inconsistent and unpredictable after the labelled number of actuations. Beyond the labelled number of actuations, propellants can release an aerosol plume that contains little or no drug, a phenomenon called tail-off [30].

### 2.2. pMDI Accessory Devices: The Spacers and Valved Holding Chambers

**Figure 2:**
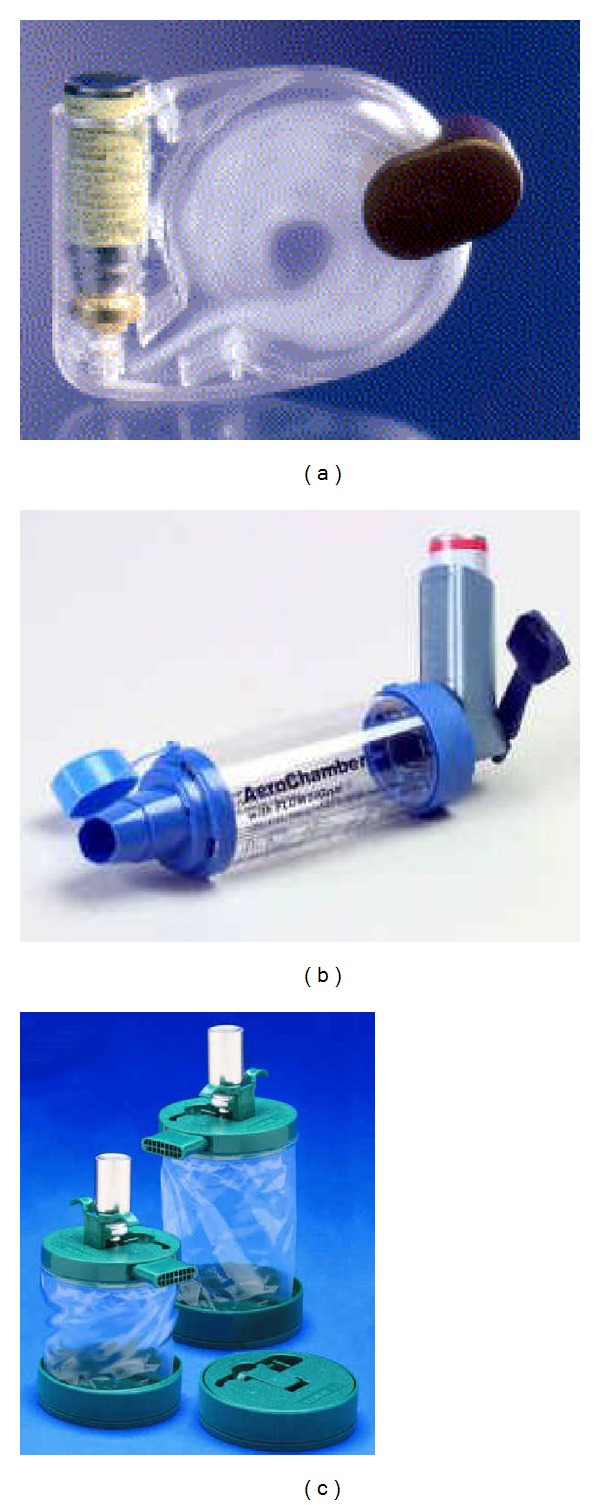
(a) The Jet open tube spacer; (b) the AeroChamber plus holding chamber; (c) the reverse-flow EZSpacer.

Although the term “spacers” is often used for all types of extension add-on devices, these devices are properly categorised as either “spacers” or “valved holding chambers.” A spacer (Figure 2) is a simple tube or extension attached to the pMDI mouthpiece with no valves to contain the aerosol plume after pMDI actuation [31]. A valved holding chamber (Figure 2) is an extension device, added onto the pMDI mouthpiece or canister, that contains a one-way valve to prevent holding the aerosol until inhalation [31]. The direction of the spray can be forward, that is, toward the mouth, or reverse, that is, away from the mouth (Figure 2). Both spacers and holding chambers constitute a volume into which the patient actuates the pMDI and from which the patient inhales reducing the need to coordinate the two manoeuvres [31]. By acting as an aerosol reservoir, these devices slow the aerosol velocity and increase transit time and distance between the pMDI actuator and the patient's mouth, allowing particle size to decrease and, consequently, increasing deposition of the aerosol particles in the lungs [31]. Moreover, because spacers trap large particles comprising up to 80% of the aerosol dose, only a small fraction of the dose is deposited in the oropharynx, thereby reducing side effects, such as throat irritation, dysphonia, and oral candidiasis, associated with medications delivered by the pMDI alone [31]. Large-volume holding chambers increase lung deposition to a greater degree than does tube spacer or small holding chamber [32–34]. Devices larger than 1 L, however, are impractical, and patients would have difficulty inhaling the compete contents [35]. A valved holding chamber fitted with an appropriate facemask is used to give pMDI drugs to neonates, young children, and elderly patients. The two key factors for optimum aerosol delivery are a tight but comfortable facemask fit and reduced facemask dead space [31, 36]. Because children have low tidal volumes and inspiratory flow rates, comfortable breathing through a facemask requires low resistance inspiratory or expiratory valves. Of note, some holding chambers incorporate a whistle that makes a sound if inspiration is too fast [36]. Training patients to ensure that the whistle does not sound assists with developing an optimal inhalation technique. Plastic bottles and cups can also be used as rudimental, home-made spacers for the administration of aerosol drugs [37–39]. In a randomized controlled trial clinical effects of salbutamol inhaled through pMDI with a home-made nonvalved spacer (500 mL mineral water plastic bottle) were compared with those when the same drug was administered by using an oxygen-driven nebuliser in children with asthma [39]. The number of children hospitalised after treatment changes in clinical score and oxygen saturation were similar in conventional and bottle spacer groups [39]. Valved holding chambers may improve the clinical effect of inhaled medications especially in patients unable to use a pMDI properly [31]. Indeed, compared to both pMDIs alone and DPIs, these devices may increase the response to short-acting *β*-adrenergic bronchodilators, even in patients with correct inhalation technique [40–43]. While spacers and valved holding chambers are good drug-delivery devices, they suffer from the obvious disadvantage of making the entire delivery system less portable and compact than a pMDI alone. The size and appearance of some spacers may detract from the appeal of the pMDI to patients, especially among the paediatric population, and negatively affect patients' compliance [31]. Furthermore, spacers are not immune from inconsistent medication delivery caused by electrostatic charge of the aerosol [44–47]. Drug deposits can build up on walls of plastic spacers and holding chambers mostly because of electrostatic charge. Aerosols remain suspended for longer periods within holding chambers that are manufactured from nonelectrostatic materials than other materials. Thus, an inhalation might be delayed for 2–5 s without a substantial loss of drug to the walls of metal or nonstatic spacers [45–47]. The electrostatic charge in plastic spacers can be substantially reduced by washing the spacer with a diluted (1 : 5000) household detergent and allowing it to drip dry [14, 48]. There is no consensus on how often a spacer should be cleaned, but recommendations range in general from once a week to once a month [12]. Multiple actuations of a pMDI into a spacer before inhalation also reduces the proportion of drug inhaled [46–50]. Five actuations of a corticosteroid inhaler into a large-volume spacer before inhalation deliver a similar dose to a single actuation into the same spacer inhaled immediately [49]. 

### 2.3. Breath-Actuated Metered-Dose Inhaler

Breath-actuated (BA) pMDIs are alternatives to conventional press-and-breath pMDIs developed to overcome the problem of poor coordination between pMDI actuation and inhalation [12, 51]. Examples of this type of device include the Autohaler (3M, St. Paul, MI) and the Easi-Breathe (Teva Pharmaceutical Industries Ltd). Breath-actuated pMDIs contain a conventional pressurised canister and have a flow-triggered system driven by a spring which releases the dose during inhalation, so that firing and inhaling are automatically coordinated [12, 51]. These inhalation devices (Table 1) can achieve good lung deposition and clinical efficacy in patients unable to use a pMDI correctly because of coordination difficulties [52]. Errors when using BApMDI are less frequent than when using a standard pMDI [17]. Increased use of BApMDIs might improve asthma control and reduce overall cost of asthma therapy compared with conventional pMDIs [53]. On the negative side (Table 1), BApMDIs do not solve cold Freon effect and would be unsuitable for a patient who has this kind of difficulty using pMDI. In addition, these devices require a relatively higher inspiratory flow than pMDI for triggering. Furthermore, oropharyngeal deposition with breath-actuated pMDIs is as high as that with CFC-pMDIs [54].

The Autohaler is a BApMDI that is available with albuterol and behlomethasone in HFA propellant. It has a manually operated lever that, when lifted, primes the inhaler through a spring-loaded mechanism, allowing the aerosol to be dispensed with an inspiratory flow of about 30 L/min. Clinical studies have demonstrated that the lung deposition of *β*-adrenergic bronchodilator administered via the Autohaler is similar to that obtained when the drug is correctly inhaled via a pMDI and greater than that resulting from conventional pMDIs in patients with poor inhalation technique [54]. Moreover, it can be used effectively by patients with poor lung function, patients with limited manual dexterity, and elderly patients [54]. The Easi-Breathe is a patient-triggered inhaler that dispenses albuterol and beclomethasone. This inhaler is primed when the mouthpiece is opened. When the patient breathes in, the mechanism is triggered and a dose is automatically released into the airstream. The inhaler can be actuated at a very low airflow rate of approximately 20 L/min, which is readily achievable by most patients [55]. Not surprisingly, practice nurses found it easier to teach and patients to use than a conventional pMDI [55]. In vitro studies have shown that particle size distribution and percentage of respirable fine particle obtained by using the Easi-Breathe device were similar to those obtained by using the conventional pMDI [56], although comparative clinical efficacy data are not yet available.

### 2.4. Dry Powder Inhalers

**Figure 3:**
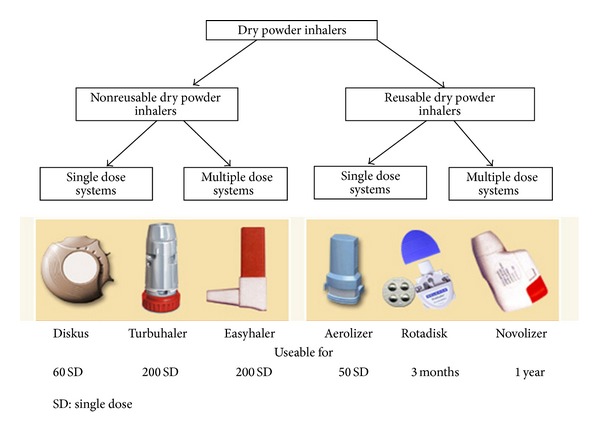
Examples of dry powder inhalers. From [57].

Modern dry powder inhalers were first introduced in 1970, and the earliest models were single-dose devices containing the powder formulation in a gelatin capsule, which the patient loaded into the device prior to use. Since the late 1980s multidose DPIs have been available, giving the same degree of convenience as a pMDI [58]. Drypowder inhalers (Figure 3) are delivery devices containing drugs in powdered formulation that have been milled to produce micronized particles in the respirable range. These delivery devices allow the particles to be deagglomerated by the energy created by the patient's own inspiratory flow [58–60]. The powdered drug can be either pure or blended with large particle size excipient (usually lactose) as a carrier powder [58–60]. The empty condition is generally apparent, alerting the patient to the need for replacement. Some DPIs, such as for the HandiHaler (Boehringer Ingelheim, D) and the Aerolizer (Novartis Pharma, CH), are singledose devices in which a capsule of powder is perforated in the device with needles fixed to pressure buttons. Other types of DPIs, such as the Diskus (GlaxoSmithKline, UK) or the Turbuhaler (AstraZeneca, Sweden), have a multidose capacity. These multidose DPIs fall into two main categories (Figure 3): these either measure the dose themselves (from a powder reservoir) or they dispense individual doses which are premetered into blisters by the manufacturer [58–60]. Turbuhaler and Diskus, respectively, are representatives of the former and latter categories, although many other different designs are presently in development. To date, new innovative DPIs are available for treatment of asthma and for delivery of a range of drugs usually given by injection, such as peptides, proteins, and vaccines. The use of DPIs is expected to increase with the phasing out of CFC production along with increased availability of drug powders and development of novel powder devices [59]. 

Generally, DPIs do have many advantages (Table 1). Dry powder inhalers are actuated and driven by patient's inspiratory flow; consequently, DPIs do not require propellants to generate the aerosol, as well as coordination of inhaler actuation with inhalation [60]. However, a forceful and deep inhalation through the DPI is needed to deaggregate the powder formulation into small respirable particles as efficiently as possible and, consequently, to ensure that the drug is delivered to the lungs [60–62]. Although most patients are capable of generating enough flow to operate a DPI efficiently [60], the need to inhale forcefully and, consequently, generate a sufficient inspiratory flow could be a problem for very young children or patients with severe airflow limitation [63]. For this reason, DPIs are not recommended for children under the age of 5 years [60]. The newer active or power-assisted DPIs incorporate battery-driven impellers and vibrating piezoelectric crystals that reduce the need for the patient to generate a high inspiratory flow rate, an advantage for many patients [59, 62]. Drug delivery to the lung ranges between 10% and 40% of the emitted dose for several marketed DPIs [60]. The physical design of the DPI establishes its specific resistance to airflow (measured as the square root of the pressure drop across the device divided by the flow rate through the device), with current designs having specific resistance values ranging from about 0.02 to 0.2 cm H_2_O/L/min) [61]. To produce a fine powder aerosol with increased delivery to the lung, a DPI that is characterised as having a low resistance requires an inspiratory flow of >90 L/min, a medium-resistance DPI requires 50–60 L/min, and a high-resistance DPI requires <50 L/min [61]. Of note, DPIs with high resistance tend to produce greater lung deposition than those with a lower resistance [61], but the clinical significance of this is not known. Based on the previous considerations, it is recommended to instruct patients to inhale forcefully from the beginning of the inspiration deeply as much as possible and to continue to inhale for as long as possible [12]. The rationale for these recommendations is that when using a DPI, inhalation should be forceful enough to disburse the micronised drug from the lactose-based carrier into a fine particle dose. However, it is not the absolute inspiratory flow that determines the fine particle dose from an inhaler but the resulting energy, which also depends on inhaler resistance. High air velocities *within* the inhaler are required for effective dispersion rather than high airflow *through* the inhaler. High airflow through the inhaler will lead to increased impaction in upper airways; thus, fast inhalation should be avoided unless a larger fine particle fraction compensates for the increased impaction. Furthermore, when using a singledose DPI, it is also recommendable to instruct patients to perform two separate inhalations for each dose [12].

Although DPIs offer advantages over pMDIs, they do have some limitations (Table 1) of design, cost-effectiveness and user-friendliness [60]. For instance, capsule-based DPIs, such as the HandiHaler and the Aerolizer, require that single doses are individually loaded into the inhaler immediately before use. This is inconvenient for some patients and does not allow direct dose counting. In addition, the inhalation manoeuvre has to be repeated until the capsule is empty, which may give rise to underdosing and to high dose variability. Other DPIs are multiple unit dose devices, such as the Diskhaler, or multidose devices, such as the Diskus and the Turbuhaler. These devices do not have any triggering mechanism which makes optimal drug delivery entirely dependent on an individual patient's uncontrolled inspiratory manoeuvre. Because of variations in the design and performance of DPIs, patients might not use all DPIs equally well. Therefore, DPIs that dispense the same drug might not be readily interchangeable [61]. Studies [59, 60] have also been shown that dose emission is reduced when a DPI is exposed to extremely low and high temperature and humidity; therefore, DPIs should be stored in a cool dry place.

A recent systematic literature review revealed that up to 90% of patients did not use their DPI correctly [64]. Common errors made by patients were lack of exhalation before inhalation, incorrect positioning and loading of the inhaler, failure to inhale forcefully and deeply through the device, and patients' failure to breath-hold after inhalation [64]. All these errors may lead to insufficient drug delivery, which adversely influences drug efficacy and may contribute to inadequate disease control [64]. It is unsurprising that such a high proportion of patients were unable to use DPIs correctly as the devices have many inherent design limitations. The Diskhaler, for example, is a multiple unit dose device as it contains a series of foil blisters on a disk. It is complicated to use, requiring eight steps to effect one correct inhalation; it has been shown that approximately 70% of patients are unable to use it correctly [64]. The disks have to be changed frequently and the device cleaned before refilling. In addition, it provides no feedback to the patient of a successful inhalation, except a sweet taste in the mouth which may simply be indicative of oral drug deposition. The Turbuhaler, a multidose reservoir device, is the most frequently prescribed DPI as it produces good deposition of the drug in the lungs provided that a sufficient (about 60 L/min) inspiratory flow has been achieved by the patients. However, approximately 80% of patients are unable to use it correctly [64]; common mistakes made by patients using this inhaler are failure to turn the base fully in both directions and failure to keep the device upright until loaded. In addition, due to its high intrinsic resistance, patients who have a reduced inspiratory flow may encounter problems using this device. The Diskus is another example of multidose device that uses a strip foil drug containing blisters. As many as 50% of patients use this DPI incorrectly, and common errors include failure or difficulty in loading the device before inhalation and exhaling into the device [64]. The Diskus has a low intrinsic resistance but, like the Turbuhaler, does not have any triggering mechanism which makes optimal drug delivery entirely dependent on an individual patient's uncontrolled inspiratory manoeuvre [64]. Additionally, as with other DPI devices employing drug blisters, incomplete emptying of the metered dose may occur, which could reduce the amount of drug delivered to the lung and hence reduce clinical efficacy [64]. 

### 2.5. Nebulisers

**Figure 4:**
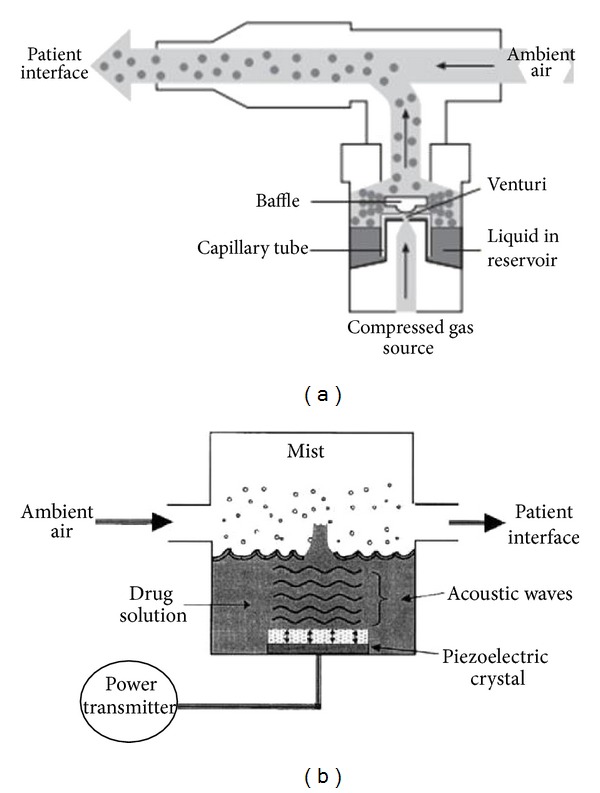
Components of a jet (a) and an ultrasonic (b) nebulisers. Modified from O'Callaghan and Barry [65].

Various types of nebulisers are available on the market, and several studies have indicated that performance varies between manufacturers and also between nebulisers from the same manufacturers [65–67]. There are two basic types (Figure 4) of nebulisers: the pneumatic or jet nebuliser and the ultrasonic nebulisers [65–67]. The jet nebulisers generate aerosol particles as a result of the impact between a liquid and a jet of high velocity gas (usually air or oxygen) in the nebuliser chamber. In a jet nebulizer, the driving gas passes through a very narrow hole from a high pressure system. At the narrow hole, the pressure falls and the gas velocity increases greatly producing a cone shaped front. This passes at high velocity over the end of a narrow liquid feed tube or concentric feeding system creating a negative pressure at this point. As a result of this fall in pressure, liquid is sucked up by the Bernoulli effect and is drawn out into fine ligaments. The ligaments then collapse into droplets under the influence of the surface tension. The majority of the liquid mass produced during this process is in the form of large (15–500 micron) nonrespirable droplets. Coarse droplets impact on baffles while smaller droplets may be inhaled or may land on internal walls returning to the reservoir for renebulisation [65–67]. The resultant large particles then impact upon baffles to generate small, respirable particles. Thus, baffle design has a critical effect on droplet size. Concentric liquid feeds minimise blockage by residual drug build-up with repeated nebulisation. A flat pick up plate may allow some nebulisers to be tilted during treatment whilst maintaining liquid flow from the reservoir. A 6–8 L/min flow and a fill volume of 4-5 mL are generally recommended, unless some nebulisers are specifically designed for different flow and a smaller or larger fill volume [68]. The volume of some unit-dose medications is suboptimal; ideally, saline should be added to bring the fill volume to 4-5 mL, but this might not be practical. The longer nebulisation time with a greater fill volume can be reduced by increasing the flow used to power the nebuliser; however, increasing the flow decreases the droplet size produced by the nebuliser. Dead volume is the volume that is trapped inside the nebulizer, and typically it is 0.5–1 mL. To reduce dead volume, clinicians and patients commonly tap the nebuliser periodically during therapy in an effort to increase nebuliser output [69]. Therapy may also be continued past the point of sputtering in an attempt to decrease the dead volume, but this is unproductive and not recommended [70]. Because of the evaporative loss within the nebuliser, the solution becomes increasingly concentrated and cools during nebulisation. 

There are four different designs of the jet nebulisers: jet nebuliser with reservoir tube, jet nebuliser with collection bag, and breath-enhanced and breath-actuated jet nebulizers [65–67]. Both the breath-enhanced and breath-actuated jet nebulisers are modifications of the “conventional” jet nebulisers specifically designed to improve their efficiency by increasing the amount of aerosol delivered to the patient with less wastage of aerosol during exhalation. The different types of jet nebulisers have different output characteristics determined by the design of the air jet and capillary tube orifices, their geometric relationship with each other and the internal baffles; for a given design the major determinant of output is the driving pressure [65–67]. The jet nebulizer with reservoir tube provides continuous aerosol during the entire breathing cycle, causing the release of aerosol to ambient air during exhalation and anytime when the patient is not breathing. Consequently, no more than 20% of the emitted aerosol is inhaled [65–67]. The jet nebulizer with collection bag generates aerosol by continuously filling a collection bag that acts as a reservoir. The patient inhales aerosol from the reservoir through a one-way inspiratory valve and exhales to the environment through an exhalation port between the one-way inspiratory valve and the mouthpiece. The breath-enhanced jet nebulizer (e.g., the PARI LC Plus, PARI gmbH) uses two one-way valves to prevent the loss of aerosol to environment. When the patient inhales, the inspiratory valve opens and aerosol vents through the nebuliser; exhaled aerosol passes through an expiratory valve in the mouthpiece. Breath-actuated jet nebulisers are designed to increase aerosol delivery to patient by means of a breath-actuated valve that triggers aerosol generation only during inspiration. Both the breath-enhanced and breath-actuated nebulisers increase the amount of inspired aerosol with shorter nebulisation time than “conventional” jet nebulisers [65]. Recently, adaptive aerosol delivery nebulisers (the HaloLite and the Prodose) have been developed to reduce the variability of the delivered dose and the waste of aerosol to the environment and to facilitate monitoring of compliance with patient therapy [71–73]. By monitoring pressure changes relative to flow over the first three breaths, these delivery systems establish the shape of the breathing pattern and then use this to provide a timed pulse of aerosol during the first 50% of each tidal inspiration. Monitoring of the breathing pattern continues throughout the delivery period, and any changes in breathing pattern are taken into account during the remainder of the delivery period. Furthermore, if no inhalation is registered, the system will cease delivery until the patient recommences breathing on the system [71–73]. Since the pulsed dose is only provided in the first 50% of each breath and the software can calculate the amount of drug given per pulse, the precise dose of drug can be delivered before the system stops [71–73]. 

Ultrasonic nebulisers use a rapidly (>1 MHz) vibrating piezoelectric crystal to produce aerosol particles [65–67]. Ultrasonic vibrations from the crystal are transmitted to the surface of the drug solution where standing waves are formed. Droplets break free from the crest of these waves and are released as aerosol. The size of droplets produced by ultrasonic nebuliser is related to the frequency of oscillation [65–67]. Although ultrasonic nebulisers can nebulise solutions more quickly than jet nebulisers, they are not suitable for suspensions and the piezoelectric crystal can heat the drug to be aerosolised. A relatively new ultrasonic nebuliser technology is represented by the vibrating mesh nebulisers [12, 74, 75]. These new-generation nebulisers are either active or passive systems. In active devices (e.g., the eFlow, PARI gmbH), the aperture plate vibrates at a high frequency and draws the solution through the apertures in the plate. In passively vibrating mesh devices (e.g., MicroAir, Omron Healthcare), the mesh is attached to a transducer horn and vibrations of the piezoelectric crystal that are transmitted via the transducer horn force the solution through the mesh to create an aerosol. The eFlow is designed to be used with either a very low residual volume to reduce drug waste or with a relatively large residual volume, so that it can be used instead of conventional jet nebulisers with the same fill volume [76]. Vibrating mesh devices have a number of advantages over other nebuliser systems: they have greater efficiency, precision and consistency of drug delivery, and are quiet and generally portable [74, 75]. However, they are also significantly more expensive than other types of nebulisers, and require a significant amount of maintenance and cleaning after each use to prevent build-up of deposit and blockage of the apertures especially when suspensions are aerosolised and to prevent colonisation by pathogens [75]. They are currently most widely used for the treatment of patients with cystic fibrosis [77].

Generally, mouthpieces are employed during nebuliser delivery. However, facemasks may be necessary for treatment of acutely dyspnoeic patients or uncooperative patients, such as infants and toddlers [78]. The facemask is not just a connector between the device and the patient. Principles of mask design are different depending on the device [78]. For example, a valved holding chamber with facemask must have a tight seal to achieve optimal lung deposition [78]. In contrast, the facemask for a nebuliser should not incorporate a tight seal but should have vent holes to reduce deposition on the face and in the eyes [79, 80]. Improvements in facemask design provide greater inhaled mass while reducing facial and ocular deposition [78]. Often, when a patient does not tolerate the facemask, practitioners employ the “blow-by” technique, which simply directs the aerosol towards the nose and mouth with the mouthpiece. However, there is no data to indicate that this is an effective method for delivering aerosol to the lungs, and therefore the use of this technique is not recommended [12].

Unlike pMDIs and DPIs, no special inhalation techniques are needed for optimum delivery with conventional nebulisers; tidal breathing with only occasional deep breaths is sufficient (Table 1). Thus, for patients who are unable to master the proper pMDI technique despite repeated instruction, the proper use of a nebuliser probably improves drug delivery. However, nebulisers have some distinct dis-advantages. Patients must load the device with medication solution for each treatment, and bacterial contamination of the reservoir can cause respiratory infection [65–67], making regular cleaning important. Also, nebuliser treatments take longer time than pMDIs and DPIs for drug administration (10–15 min for a jet nebuliser, 5 min for an ultrasonic or mesh nebuliser). Although they are relatively portable, typical jet nebuliser must be plugged into a wall outlet or power adaptor and thus cannot be used easily in transit. 

### 2.6. Soft Mist Inhalers

**Figure 5:**
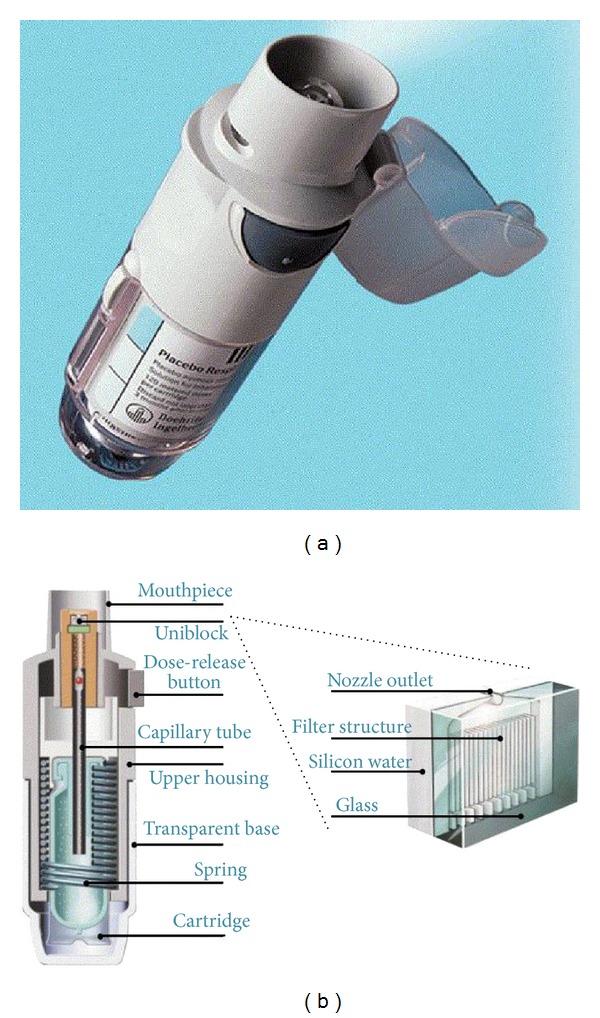
The Respimat soft mist inhaler. From [81].

The development of soft mist inhalers (SMIs) has opened up new opportunities for inhaled drug delivery. Technically, these inhalation devices do fall within the definition of a nebuliser, as they transform aqueous liquid solution to liquid aerosol droplets suitable for inhalation. However, at variance with the traditional nebuliser designs, SMIs are hand-held multidose devices that have the potential to compete with both pMDIs and DPIs in the portable inhaler market. At the present, the only SMI currently marketed in some European countries is the Respimat inhaler (Boehringer Ingelheim, Figure 5). This device does not require propellants since it is powered by the energy of a compressed spring inside the inhaler. Individual doses are delivered via a precisely engineered nozzle system as a slow-moving aerosol cloud (hence the term “soft mist”) [81]. Scintigraphic studies have shown that, compared to a CFC-based pMDI, lung deposition is higher (up to 50%) and oropharyngeal deposition is lower [81]. Respimat is a “press and breathe” device, and the correct inhalation technique closely resembles that used with a pMDI. However, although coordination between firing and inhaling is required, the aerosol emitted from Respimat is released very slowly, with a velocity of approximately four times less than that observed with a CFC-driven pMDI [81]. This greatly reduces the potential for drug impaction in the oropharynx. In addition, the relatively long duration over which the dose is expelled from Respimat (about 1.2 s compared with 0.1 s from pMDIs) would be expected to greatly reduce the need to coordinate actuation and inspiration, thus improving the potential for greater lung deposition. Although Respimat has been used relatively little in clinical practice to date, clinical trials seem to confirm that drugs delivered by the Respimat are effective in correspondingly smaller doses in patients with obstructive airway disease [82].

## 3. Choice of an Inhaler Device for Asthma Therapy

Drug choice is usually the first step in prescribing inhaled therapy for asthma and, together with availability and reimbursement criteria, dictates the inhaler deliver options. The next two steps, choice of inhaler device type and patient training in the use of the inhaler, are hampered by the lack of robust evidence or effective tools to aid healthcare professionals [9, 10, 83]. Meta-analysis regarding the selection of aerosol delivery systems for acute asthma concluded that short-acting beta agonists delivered via either nebuliser or pMDI with handling chamber are essentially equivalent [84–88]. More than 100 inhaled device-drug combinations are currently available for treatment of asthmatic patients [57]. The number is likely to increase with the development of analogue inhaled drugs delivered by relatively low-cost pMDIs and DPIs. Consequently, the level of confusion experienced by clinicians, nurses, and pharmacists when trying to choose the most appropriate device for each patient is increased. Thus, physicians' experience is amongst the most important factors which influence decision making for inhaler choice in asthma therapy. In fact, inhalers are often prescribed on an empirical basis rather than on an evidence-based approach. Following their own experience, doctors are much more likely to prescribe the same old inhaler which they have always prescribed rather than new, improved inhalers entering the market. 

Current asthma management guidelines give some guidance on the class of inhaler to prescribe to children, but they offer nonspecific advice regarding inhaler choice for adult patients. The GINA guidelines [1] recommend pMDIs with spacer and facemask for children younger than 4 years (or pMDIs with spacer and mouthpiece for those aged 4–6 years) and, in addition to pMDIs alone, DPIs or BAMDIs, for children older than 6 years. However, for adults, the same guidelines state that inhalers should be portable and simple to use, should not require an external power source, require minimal cooperation and coordination, and have minimal maintenance requirements [1]. The British Thoracic Society guidelines [2] also include patient's preference and abilities to use the device correctly. However, this advice relating to patient preference is not supported by any evidence that patients will correctly use an inhaler that they like. 

Criteria to be considered when choosing an inhaler device differ depending on the audience addressed [89]. From the viewpoint of the inhalation technologist, consistent and safe dosing, sufficient drug deposition, and clinical effect guide the inhaler choice. The patient's ability to inhale through the device, the intrinsic airflow resistance of the device, and the degree of dependence of drug release on inspiratory airflow variability are all important determinants when considering constancy of dosing [89]. From the point of view of the clinician, clinical efficacy and safety should be the most important determinants to consider when choosing an inhaler [89]. However, in the real word clinical efficacy must be balanced against cost-effectiveness, and inhalers with insufficient performance may be prescribed simply because they are cheap. Patients' preferences and acceptance of the inhaler should also be considered when deciding on a specific inhaler since these will have major implications for compliance. 

Several general principles of inhaler selection and use have recently been addressed in an evidence-based systematic review by a joint committee of the American College of Chest Physicians and the American College of Asthma, Allergy and Immunology [13]. The bottom line of this document was that each of the aerosol devices can work equally well in various clinical settings with patients who can use these devices properly [13]. In addition, pMDIs are convenient for delivering a wide variety of drugs to a broad spectrum of patients. For patients who have trouble coordinating inhalation with inhaler actuation, the use of spacer may obviate this difficulty, though most of these devices are cumbersome to store and transport [13]. The use of spacer, however, is mandatory for infants and young children. Dry powder inhalers are usually easier for patients to handle than pMDIs, and a growing number of drug types are available in several DPI formats [13]. The key issue for dry powder inhalation is the minimum inspiratory flow rate below which deagglomeration is inefficient, resulting in a reduced drug delivered dose. The most ill patients and the very young may not be candidates for a DPI. A nebuliser could be used as an adequate alternative to pMDI with a spacer by almost any patient in a variety of clinical settings from the home to the intensive care unit [13]. However, nebulisers are more expensive, cumbersome, and relatively time-consuming to use compared to hand-held inhalers. These attributes should limit the use of nebulisers whose effect can be matched by hand-held devices in almost all clinical settings. The findings of this document should not be interpreted to mean that the device choice for a specific patient does not matter. Rather, the study simply says that each of the devices studied can work equally well in patients who can use them correctly. However, this evidence-based systematic review does not provide much information about who is likely to use one device or another properly nor does it address many other considerations that are important for choosing a delivery device for a specific patient in a specific clinical situation. These include the patient's ability to use the device, patient preference, and the availability of equipment and cost. 

More recently, Chapman and coworkers [90] proposed an algorithm approach to inhaler selection that considers patient's ability to generate an inspiratory flow rate >30 L/min to coordinate inhaler actuation and inspiration and to prepare and actuate the device (Table 2). 

When choosing an inhaler for children, it is essential that the individual child receives appropriate instructions and training necessary for the management of the disease [91]. Furthermore, the child should be prescribed the correct medication tailored to the severity of the disease, and, most importantly, the prescribed inhaler should suit the individual needs and preference of the child [91]. Contrary to general opinion, using an inhaler may be difficult for children [91]; many children with asthma use their inhaler incorrectly which may result in unreliable drug delivery, even after instruction and training for correct inhalation. In addition, previous inhalation instruction may be forgotten and, therefore, training should be repeated regularly to maintain correct inhalation technique in children with asthma [91].

## 4. Education and Instruction

Successful asthma management is 10% medication and 90% education [92]. Asthma education empowers patients to manage their disease and increases their awareness of danger signs [93]. Patients with a positive attitude towards controlling their asthma are more likely to adhere to therapy [94]. Regular medical review provides an opportunity to raise patients' expectations, helps them understand how to monitor their asthma, and increases awareness of possible factors, such as poor inhaler technique, that may prevent them from attaining control [93]. A key challenge in many practice situations is the allocation of personnel and time for patient training in inhaler technique, although the upfront investment in time to properly train could later save time, resources, and adverse patient impact by preventing uncontrolled asthma because of poor inhaler technique. The conventional wisdom is that training patients to use inhalers is time-consuming. However, in one study, training sessions provided by pharmacists took an average of only 2.5 min and were shown to improve asthma outcomes [95]. The “trainer” must know the proper technique, including refinements to optimise inhaler therapy for each device type prescribed. However, the healthcare professionals involved often have not mastered inhalation technique themselves [96, 97] and are not sufficiently aware of handling difficulties with devices other than pMDIs [98]. Furthermore, only 2 of the 40 medical textbooks include a simple list of steps to properly use a pMDI [11]. For this reason, studies have examined educational interventions designed to “train the trainer” and improve healthcare professional inhaler competence. It has been demonstrated that a single education session improves medical residents' inhaler knowledge and skills [99]. Another study demonstrated that pharmacists who participate in a single-session education workshop showed significantly better knowledge and skills than a control group and that this knowledge was retained at a high level [100]. The best person to provide inhaler training (physician, nurse, or pharmacist) will vary by practice situation. Another option is to enlist the aid of lay educators (e.g., other patients) to provide support and training. In all cases, adequate time and resources must be allotted for the training sessions. Successful training in inhaler technique depends upon effective communication of proper technique and its purpose and monitoring to ensure that the skills have been learned and retained [101]. Of all the training approaches possible, personal or small group demonstration has so far proven most effective [102, 103]. Other training methods for inhaler use include written indications, illustrations, audio-visual demonstrations, and internet-based, interactive, multimedia tutorials, the latter representing a promising, new low-cost and time-saving mechanism for educating both patients and healthcare professionals [104]. However, their value must not be overestimated, as a substantial proportion of patients still have incorrect inhalation technique despite several training sessions [105] Periodic retraining is needed as inhaler technique deteriorates with time [104, 106]. Special provision should be made for the elderly, who may have more trouble learning good inhaler technique and a greater tendency to forget it, while small children may require a particular teaching environment to hold their attention [107, 108]. Intuitively, therapeutic success will be more likely if patients are prescribed a device that they have chosen, are happy with, and can use well. Although use of a single type of device to deliver all medications is not always practicable it is preferable since coping with a variety of devices increases the likelihood of error [109]. 

A visual evaluation by the healthcare professionals is subjective but important in assessing inhaler preparation and the mechanics of inhaler handling by the patient. Indeed, in real life, patients make many errors with their usual inhalation device that may negate the benefits observed in clinical trials. A checklist to identify critical errors, which are those comprising treatment efficacy, could be applied here, as outlined by Molimard and Le Gros [110]. Examples of currently available tools to objectively check and maintain the correct inhalation pattern include the Aerosol Inhalation Monitor (Vitalograph Ltd., Buckingham, UK) and 2Tone Trainer (Canday Medical Ltd., Newmarket, UK) for MDIs and the In-Check Dial (Clement Clarke International, Harlow, UK) for DPIs [111, 112]. These tools can provide an objective evaluation of the inhalation profile but cannot assess the patient's preparation and handling of their device.

## 5. ADMIT Recommendations 

Many physicians in Europe are fully aware of the difficulties that patients have using prescribed inhaler devices correctly and the negative impact that this may have on asthma control. The *Aerosol Drug Management Improvement Team* (ADMIT), a consortium of European respiratory physicians (respiratory specialists, general practitioners, and paediatricians) with a common interest in promoting excellent delivery of inhaled drugs, was formed with the remit of examining ways to improve treatment of obstructive airway disease in Europe [57]. ADMIT recommends that instructions for correct inhalation technique for each inhaler device currently on the market should be compiled by an Official Board with instructions made readily accessible on the web. Local asthma associations and patient groups could also be involved in promoting the importance and teaching and reinforcing of correct inhalation technique. Information could be disseminated by the use of dedicated literature, school visits by healthcare professionals, pharmacists, and through patient advocacy groups. Other ADMIT recommendations are summarised as follows.

Recommendations from the Aerosol Drug Management Improvement Team (ADMIT) for the choice and correct usage of inhalers. (DPI, dry powder inhaler; PIF, peak inspiratory flow; pMDI, pressurised metered-dose inhaler. Modified from Crompton et al. [8].)

- Inhalers should be matched to the patient as much as possible.
- In young children, pMDIs should be always used with a spacer device.
- An alternative to a pMDI should be considered in elderly patients with a minimental test score <23/30 or an ideomotor dyspraxia score <14/20 as they are unlikely to have correct inhalation technique through a pMDI.
- The patient's PIF values should be considered before DPI prescription. Those patients with severe airflow obstruction, children, and the elderly would benefit from an inhaler device with a low airflow resistance.
- Before prescribing a DPI, check that the patient can inhale deeply and forcibly at the start of the inspiration as airflow profile affects particle size produced and hence drug deposition and efficacy.
- Where possible, one patient should have one type of inhaler.
- Establish an Official Board to compile instructions for correct inhalation technique for each inhaler device currently on the market.
- Instructions for correct inhaler use should be made readily accessible on a dedicated web site.
- Training in correct inhalation technique is essential for patients and healthcare professionals.
- Inhalation technique should be checked and reinforced at regular intervals.
- Teaching correct inhalation techniques should be tailored to the patient's needs and preferences: group instruction in correct inhalation technique appears to be more effective than personal one-to-one instruction and equally effective like video instruction; younger patients may benefit more from multimedia teaching methods; elderly patients respond well to one-to-one tuition.

**Figure 6:**
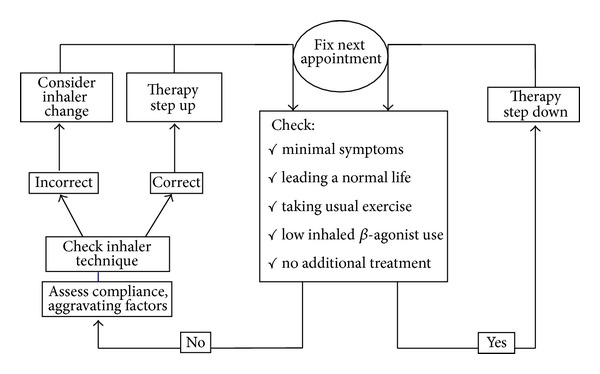
Asthma therapy adjustment flow chart. From [8].

The ADMIT has also proposed a practical algorithm (Figure 6) in order to improve the instruction given to the patient regarding optimal use of their inhalers. At each consultation of the patient, the physician should establish the patient's level of symptoms and control, ideally using a composite measure such as the GINA control assessment [1], and if well controlled for at least 3 months, therapy should be stepped down gradually according to treatment guidelines. Conversely, if the patient answers “no” to any of these checklist questions then compliance and aggravating (trigger) factors should be assessed. Most importantly, inhalation technique should be assessed. If the patient is unable to use a particular inhaler correctly despite repeated attempts, a change in inhaler device should be considered. In the cases where ongoing uncontrolled asthma persists in the face of correct inhaler technique, then asthma therapy should be stepped up according to the treatment guidelines and another appointment scheduled in order to check symptoms.

## 6. Conclusions

The prevalence of asthma is continuing to rise throughout the world, particularly amongst children. Despite the implementation of both national and international guidelines and the widespread availability of effective pharmacological therapy, asthma is frequently uncontrolled and still may cause death. The reasons for this anomaly are numerous. First, the guidelines themselves are complex and too long for most physicians to absorb and utilise excessive length. Secondly, patients frequently do not adhere to their treatment regimen for a variety of reasons including incorrect use of inhaler and underestimation of disease severity. Indeed, asthma severity is often misclassified in the first instance and inappropriate or insufficient therapy prescribed. Finally, although guidelines agree on the most appropriate therapy to control asthma, the method by which this therapy is delivered to the lungs is often lacking in detail.

To date, advancement in asthma management has been pharmacologically driven rather than device driven. Since it is likely that in the future inhaled bronchodilators and corticosteroids will remain the cornerstone of asthma therapy, development of inhaler devices may become more important than development of new drugs. In the past 10–15 years, several innovative developments have advanced the field of inhaler design. Although many inhalers incorporate features providing efficient aerosol delivery for asthma treatment, there is no perfect inhaler and each has advantages and disadvantages, but there is increasing recognition that a successful clinical outcome is determined as much by choice of an appropriate inhaler device as by the drugs that go in them. Drug delivery from all inhaler devices depends on how the patient prepares the device and then inhales from it. Problems with drug delivery have been identified due to inappropriate use of inhaler devices, particularly pMDIs where patients need to coordinate inhaler activation with inspiration. However, as inhalation is likely to remain the delivery route of choice for the foreseeable future, there is a need to develop inhaler devices which are easy to use and deliver a consistent dose of drug to the lungs which may improve patient compliance with treatment leading to better control of asthma. There is evidence that a patient is most likely to use correctly an inhaler that he or she prefers, and each patient's choice of device will be determined by individual perceptions of how its advantages and disadvantages balance out. This decision could be quite different to the judgment of a prescriber or a formulator, who may give more weight to technical points. Choice of an inhaler device should therefore take into account the likelihood that patients will be able to use a particular device correctly, cost-effectiveness, preference, and likely compliance.

Continued and repeated education of both healthcare professionals and patients in correct inhalation technique is essential, and the results are checked at regular intervals by a member of medical staff. Substantial changes in educational efforts are clearly required and should be particularly addressed towards the general practitioner and asthma nurse who in turn teach patients how to use their inhaler correctly. Finally, it is important to remember that continually changing inhaler devices which deliver the same drug is not the answer, as patients lose confidence in both the device and the drug and compliance with therapy drops. An inhaler should only be prescribed with the absolute certainly that the patient can use it correctly. It should be stressed that once a patient is familiar and stabilised on one type of inhaler, they should not be switched to new devices without their involvement and without follow-up education on how to use the device properly. A recent study has shown that asthma control deteriorates if an inhaler is substituted for a different device at the prescribing or dispensing stage without involving the patient [113]. Prescribers should be especially vigilant on this point in order to avoid changes to the type of device their patients receive through the pharmacy.

## Figures and Tables

**Table 1:** Major components, advantages, and disadvantages of inhaler devices.

Inhaler	Formulation	Metering system	Advantages	Disadvantages
pMDI	Drug suspended or dissolved in propellant (with surfactant and cosolvent)	Metering valve and reservoir	Portable and compact Multidose device Relatively cheap Cannot contaminate contents Available for most inhaled medications	Contains propellants Not breath-actuated Many patients cannot use it correctly High oropharyngeal deposition
pMDI + spacer			Easier to coordinate Large drug doses delivered more conveniently Less oropharyngeal deposition Higher lung deposition than a pMDI	Less portable than pMDI Plastic spacers may acquire static charge Additional cost to pMDI

BA-MDI	Drug suspended in propellant	Metering valve and reservoir	Portable and compact Multidose device Breath-actuated (no coordination needed) Cannot contaminate contents	Contains propellants “Cold Freon” effect Requires moderate inspiratory flow to be triggered

DPI	Drug blend in lactose, drug alone, drug/excipient particles	Capsules, blisters, multidose blister packs reservoirs	Portable and compact Breath-actuated (no coordination needed) Does not contain propellants	Requires a minimum inspiratory flow May not appropriate for emergency situations Many patients cannot use it correctly Most types are moisture sensitive

SMI (Respimat)	Aqueous solution or suspension	Unit dose blisters or reservoirs	Portable and compact Multidose device High lung deposition Does not contain propellants	Not breath-actuated Not currently available in most countries Relatively expensive

Nebulisers	Aqueous solution or suspension	Nebule dispensed into reservoir chamber of nebulizer	May be used at any age No specific inhalation technique required Vibrating mesh is portable and does not require an outside energy source May dispense drugs not available with pMDIs or DPIs	Jet and ultrasonic nebulisers require an outside energy source Treatment times can be long Performance varies between nebulisers Jet nebulisers cannot aerosolise a certain volume of solution Risk of bacterial contamination Newer nebulisers are expensive

pMDI: pressurised metered-dose inhalers; BA-MDI: breath-actuated metered-dose inhaler; DPI: dry-powder inhaler; SMI: soft mist inhaler.

**Table 2:** Choice of inhaler devices according to the patient's inspiratory flow and ability to coordinate inhaler actuation and inhalation. Modified from Chapman et al. [90].

Good hand-lung coordination		Poor hand-lung coordination	
Inspiratory flow > 30 L/min	Inspiratory flow < 30 L/min	Inspiratory flow > 30 L/min	Inspiratory flow < 30 L/min
pMDI	pMDI	pMDI + spacer	pMDI + spacer
BAMDI	Nebuliser	BAMDI	Nebuliser
DPI	SMI	DPI	SMI
Nebuliser		Nebuliser	
SMI		SMI	

pMDI: pressurised metered-dose inhalers; BAMDI: breath-actuated metered-dose inhaler; DPI: dry powder inhaler; SMI: soft mist inhaler.

## References

[1] Global Initiative for Asthma. Global strategy for asthma management and prevention. http://www.asthmacure.com/.

[2] British Thoracic Society Scottish Intercollegiate Guidelines Network (2008). British Guideline on the Management of Asthma. *Thorax*.

[3] Rabe KF, Vermeire PA, Soriano JB, Maier WC (2000). Clinical management of asthma in 1999: the Asthma Insights and Reality in Europe (AIRE) study. *European Respiratory Journal*.

[4] Gruffydd-Jones K (2002). Measuring pulmonary function in practice. *Practitioner*.

[5] Barnes PJ, Jonsson B, Klim JB (1996). The costs of asthma. *European Respiratory Journal*.

[6] Chen H, Gould MK, Blanc PD (2007). Asthma control, severity, and quality of life: quantifying the effect of uncontrolled disease. *Journal of Allergy and Clinical Immunology*.

[7] O'Byrne PM, Pedersen S, Schatz M (2013). The poorly explored impact of uncontrolled asthma. *Chest*.

[8] Crompton GK, Barnes PJ, Broeders M (2006). The need to improve inhalation technique in Europe: a report from the Aerosol Drug Management Improvement Team. *Respiratory Medicine*.

[9] Price D, Bosnic-Anticevich S, Briggs A (2013). Inhaler competence in asthma: common errors, barriers to use and recommended solutions. *Respiratory Medicine*.

[10] Papi A, Haughney J, Virchow JC, Roche N, Palkonen S, Price D (2011). Inhaler devices for asthma: a call for action in a neglected field. *European Respiratory Journal*.

[11] Fink JB, Rubin BK (2005). Problems with inhaler use: a call for improved clinician and patient education. *Respiratory Care*.

[12] Laube BL, Janssens HM, De Jongh FHC (2011). What the pulmonary specialist should know about the new inhalation therapies. *European Respiratory Journal*.

[13] Dolovich MB, Ahrens RC, Hess DR (2005). Device selection and outcomes of aerosol therapy: evidence-based guidelines: American College of Chest Physicians/American College of Asthma, Allergy, and Immunology. *Chest*.

[14] Sanchis J, Corrigan C, Levy ML, Viejo JL (2013). Inhaler devices—from theory to practice. *Respiratory Medicine*.

[15] Dolovich MB, Dhand R (2011). Aerosol drug delivery: developments in device design and clinical use. *The Lancet*.

[16] Hess DR (2008). Aerosol delivery devices in the treatment of asthma. *Respiratory Care*.

[17] Lenney J, Innes JA, Crompton GK (2000). Inappropriate inhaler use: assessment of use and patient preference of seven inhalation devices. *Respiratory Medicine*.

[18] Lavorini F, Corrigan CJ, Barnes PJ (2011). Retail sales of inhalation devices in European countries: so much for a global policy. *Respiratory Medicine*.

[19] Hendeles L, Colice GL, Meyer RJ (2007). Withdrawal of albuterol inhalers containing chlorofluorocarbon propellants. *The New England Journal of Medicine*.

[20] Ross DL, Gabrio BJ (1999). Advances in metered dose inhaler technology with the development of a chlorofluorocarbon-free drug delivery system. *Journal of Aerosol Medicine*.

[21] Ganderton D, Lewis D, Davies R, Meakin B, Brambilla G, Church T (2002). Modulite: a means of designing the aerosols generated by pressurized metered dose inhalers. *Respiratory Medicine*.

[22] Leach CL (2005). The CFC to HFA transition and its impact on pulmonary drug development. *Respiratory Care*.

[23] Gabrio BJ, Stein SW, Velasquez DJ (1999). A new method to evaluate plume characteristics of hydrofluoroalkane and chlorofluorocarbon metered dose inhalers. *International Journal of Pharmaceutics*.

[24] Acerbi D, Brambilla G, Kottakis I (2007). Advances in asthma and COPD management: delivering CFC-free inhaled therapy using Modulite technology. *Pulmonary Pharmacology and Therapeutics*.

[25] Dhillon S, Keating GM (2006). Beclometasone dipropionate/formoterol: in an HFA-propelled pressurised metered-dose inhaler. *Drugs*.

[26] Newman SP, Pavia D, Clarke SW (1981). How should a pressurized *β*-adrenergic bronchodilator be inhaled?. *European Journal of Respiratory Diseases*.

[27] Crompton GK (2004). How to achieve good compliance with inhaled asthma therapy. *Respiratory Medicine*.

[28] Crompton GK (1990). The adult patient’s difficulties with inhalers. *Lung*.

[29] Giraud V, Roche N (2002). Misuse of corticosteroid metered-dose inhaler is associated with decreased asthma stability. *European Respiratory Journal*.

[30] Schultz RK (1995). Drug delivery characteristics of metered-dose inhalers. *Journal of Allergy and Clinical Immunology*.

[31] Lavorini F, Fontana GA (2009). Targeting drugs to the airways: the role of spacer devices. *Expert Opinion on Drug Delivery*.

[32] Newman SP, Newhouse MT (1996). Effect of add-on devices for aerosol drug delivery: deposition studies and clinical aspects. *Journal of Aerosol Medicine*.

[33] Barry PW, O’Callaghan C (1996). Inhalational drug delivery from seven different spacer devices. *Thorax*.

[34] Barry PW, O’Callaghan C (1995). The optimum size and shape of spacer devices for inhalational therapy. *Journal of Aerosol Medicine*.

[35] Newman SP (2004). Spacer devices for metered dose inhalers. *Clinical Pharmacokinetics*.

[36] Mitchell JP, Nagel MW (2007). Valved holding chambers (VHCs) for use with pressurised metered-dose inhalers (pMDIs): a review of causes of inconsistent medication delivery. *Primary Care Respiratory Journal*.

[37] Zar HJ, Asmus MJ, Weinberg EG (2002). A 500-ml plastic bottle: an effective spacer for children with asthma. *Pediatric Allergy and Immunology*.

[38] Taylor SA, Asmus MJ, Liang J, Coowanitwong I, Vafadari R, Hochhaus G (2003). Performance of a corticosteroid inhaler with a spacer fashioned from a plastic cold-drink bottle: effects of changing bottle volume. *Journal of Asthma*.

[39] Duarte M, Camargos P (2002). Efficacy and safety of a home-made non-valved spacer for bronchodilator therapy in acute asthma. *Acta Paediatrica, International Journal of Paediatrics*.

[40] Fontana GA, Lavorini F, Chiostri M, Castellani W, Boddi V, Pistolesi M (1999). Large and small airway responses to procaterol hydrochloride administered through different extension devices in asthmatic patients. *Journal of Aerosol Medicine*.

[41] Lavorini F, Geri P, Luperini M (2004). Clinical and functional responses to salbutamol inhaled via different devices in asthmatic patients with induced bronchoconstriction. *British Journal of Clinical Pharmacology*.

[42] Lavorini F, Geri P, Mariani L (2006). Speed of onset of bronchodilator response to salbutamol inhaled via different devices in asthmatics: a bioassay based on functional antagonism. *British Journal of Clinical Pharmacology*.

[43] Lavorini F, Geri P, Camiciottoli G, Pistolesi M, Fontana GA (2008). Agreement between two methods for assessing bioequivalence of inhaled salbutamol. *Pulmonary Pharmacology and Therapeutics*.

[44] Wildhaber JH, Devadason SG, Eber E (1996). Effect of electrostatic charge, flow, delay and multiple actuations on the in vitro delivery of salbutamol from different small volume spacers for infants. *Thorax*.

[45] O’Callaghan C, Lynch J, Cant M, Robertson C (1993). Improvement in sodium cromoglycate delivery from a spacer device by use of an antistatic lining, immediate inhalation, and avoiding multiple actuations of drug. *Thorax*.

[46] Barry PW, O’Callaghan C (1995). The effect of delay, multiple actuations and spacer static charge on the in vitro delivery of budesonide from the Nebuhaler. *British Journal of Clinical Pharmacology*.

[47] Clark DJ, Lipworth BJ (1996). Effect of multiple actuations, delayed inhalation and antistatic treatment on the lung bioavailability of salbutamol via a spacer device. *Thorax*.

[48] Barry PW, Robertson CF, O’Callaghan C (1993). Optimum use of a spacer device. *Archives of Disease in Childhood*.

[49] Barry PW, O’Callaghan C (1994). Multiple actuations of salbutamol MDI into a spacer device reduce the amount of drug recovered in the respirable range. *European Respiratory Journal*.

[50] Rau JL, Restrepo RD, Deshpande V (1996). Inhalation of single vs multiple metered-dose bronchodilator actuations from reservoir devices: an in vitro study. *Chest*.

[51] Crompton G, Duncan J (1989). Clinical assessment of a new breath-actuated inhaler. *Practitioner*.

[52] Gross G, Cohen RM, Guy H (2003). Efficacy response of inhaled HFA-albuterol delivered via the breath-actuated Autohaler inhalation device is comparable to dose in patients with asthma. *Journal of Asthma*.

[53] Price D, Thomas M, Mitchell G, Niziol C, Featherstone R (2003). Improvement of asthma control with a breath-actuated pressurised metred dose inhaler (BAI): a prescribing claims study of 5556 patients using a traditional pressurised metred dose inhaler (MDI) or a breath-actuated device. *Respiratory Medicine*.

[54] Newman SP, Weisz AWB, Talaee N, Clarke SW (1991). Improvement of drug delivery with a breath actuated pressurised aerosol for patients with poor inhaler technique. *Thorax*.

[55] Price DR, Pearce L, Powell SR, Shirley J, Sayers MK (1999). Handling and acceptability of the Easi-Breathe device compared with a conventional metered dose inhaler by patients and practice nurses. *International Journal of Clinical Practice*.

[56] Lipworth BJ, Clark DJ (1997). Lung delivery of salbutamol given by breath activated pressurized aerosol and dry powder inhaler devices. *Pulmonary Pharmacology and Therapeutics*.

[57] http://www.admit-online.info/.

[58] Newman SP, Busse WW (2002). Evolution of dry powder inhaler design, formulation, and performance. *Respiratory Medicine*.

[59] Islam N, Gladki E (2008). Dry powder inhalers (DPIs)-A review of device reliability and innovation. *International Journal of Pharmaceutics*.

[60] Atkins PJ (2005). Dry powder inhalers: an overview. *Respiratory Care*.

[61] Azouz W, Chrystyn H (2012). Clarifying the dilemmas about inhalation techniques for dry powder inhalers: integrating science with clinical practice. *Primary Care Respiratory Journal*.

[62] Islam N, Cleary MJ (2012). Developing an efficient and reliable dry powder inhaler for pulmonary drug delivery—a review for multidisciplinary researchers. *Medical Engineering and Physics*.

[63] Janssens W, VandenBrande P, Hardeman E (2008). Inspiratory flow rates at different levels of resistance in elderly COPD patients. *European Respiratory Journal*.

[64] Lavorini F, Magnan A, Christophe Dubus J (2008). Effect of incorrect use of dry powder inhalers on management of patients with asthma and COPD. *Respiratory Medicine*.

[65] O’Callaghan C, Barry PW (1997). The science of nebulised drug delivery. *Thorax*.

[66] Hess DR (2000). Nebulizers: principles and performance. *Respiratory Care*.

[67] Boe J, Dennis JH, O’Driscoll BR (2001). European respiratory society guidelines on the use of nebulizers. *European Respiratory Journal*.

[68] Hess D, Fisher D, Williams P, Pooler S, Kacmarek RM (1996). Medication nebulizer performance: effects of diluent volume, nebulizer flow, and nebulizer brand. *Chest*.

[69] Everard ML, Evans M, Milner AD (1994). Is tapping jet nebulisers worthwhile?. *Archives of Disease in Childhood*.

[70] Malone RA, Hollie MC, Glynn-Barnhart A, Nelson HS (1993). Optimal duration of nebulized albuterol therapy. *Chest*.

[71] Nikander K (1997). Adaptive aerosol delivery: the principles. *European Respiratory Review*.

[72] Denyer J (1997). Adaptive aerosol delivery in practice. *European Respiratory Review*.

[73] Van Dyke RE, Nikander K (2007). Delivery of iloprost inhalation solution with the halolite, prodose, and I-neb adaptive aerosol delivery systems: an in vitro study. *Respiratory Care*.

[74] Skaria S, Smaldone GC (2010). Omron NE U22: comparison between vibrating mesh and jet nebulizer. *Journal of Aerosol Medicine and Pulmonary Drug Delivery*.

[75] Dhand R (2002). Nebulizers that use a vibrating mesh or plate with multiple apertures to generate aerosol. *Respiratory Care*.

[76] Coates AL, Green M, Leung K (2011). A comparison of amount and speed of deposition between the PARI LC STAR jet nebulizer and an investigational eFlow nebulizer. *Journal of Aerosol Medicine and Pulmonary Drug Delivery*.

[77] Naehrig S, Lang S, Schiffl H, Huber RM, Fischer R (2011). Lung function in adult patients with cystic fibrosis after using the eFlow rapid for one year. *European Journal of Medical Research*.

[78] Smaldone GC, Sangwan S, Shah A (2007). Facemask design, facial deposition, and delivered dose of nebulized aerosols. *Journal of Aerosol Medicine*.

[79] Erzinger S, Schueepp KG, Brooks-Wildhaber J, Devadason SG, Wildhaber JH (2007). Facemasks and Aerosol delivery in vivo. *Journal of Aerosol Medicine*.

[80] Sangwan S, Gurses BK, Smaldone GC (2004). Facemasks and facial deposition of aerosols. *Pediatric Pulmonology*.

[81] Dalby R, Spallek M, Voshaar T (2004). A review of the development of Respimat Soft Mist Inhaler. *International Journal of Pharmaceutics*.

[82] Kässner F, Hodder R, Bateman ED (2004). A review of ipratropium bromide/fenoterol hydrobromide (Berodual) delivered via Respimat Soft Mist Inhaler in patients with asthma and chronic obstructive pulmonary disease. *Drugs*.

[83] Haughney J, Price D, Barnes NC, Virchow JC, Roche N, Chrystyn H (2010). Choosing inhaler devices for people with asthma: current knowledge and outstanding research needs. *Respiratory Medicine*.

[84] Cates CC, Bara A, Crilly JA, Rowe BH (2003). Holding chambers versus nebulisers for beta-agonist treatment of acute asthma. *Cochrane Database of Systematic Reviews*.

[85] Cates C (2003). Spacers and nebulisers for the delivery of beta-agonists in non-life-threatening acute asthma. *Respiratory Medicine*.

[86] Brocklebank D, Ram F, Wright J (2001). Comparison of the effectiveness of inhaler devices in asthma and chronic obstructive airways disease: a systematic review of the literature. *Health Technology Assessment*.

[87] Turner MO, Patel A, Ginsburg S, FitzGerald JM (1997). Bronchodilator delivery in acute airflow obstruction: a meta-analysis. *Archives of Internal Medicine*.

[88] Amirav I, Newhouse MT (1997). Metered-dose inhaler accessory devices in acute asthma: efficacy and comparison with nebulizers. A literature review. *Archives of Pediatrics and Adolescent Medicine*.

[89] Christian Virchow J (2005). What plays a role in the choice of inhaler device for asthma therapy?. *Current Medical Research and Opinion*.

[90] Chapman KR, Voshaar TH, Virchow JC (2005). Inhaler choice in primary practice. *European Respiratory Review*.

[91] Brand PL (2005). Key issues in inhalation therapy in children. *Current Medical Research and Opinion*.

[92] Fink JB (2005). Inhalers in asthma management: is demonstration the key to compliance?. *Respiratory Care*.

[93] Gibson PG, Ram FSF, Powell H (2003). Asthma education. *Respiratory Medicine*.

[94] Bailey WC, Richards JM, Brooks CM, Soong S, Windsor RA, Manzella BA (1990). A randomized trial to improve self-management practices of adults with asthma. *Archives of Internal Medicine*.

[95] Basheti IA, Reddel HK, Armour CL, Bosnic-Anticevich SZ (2007). Improved asthma outcomes with a simple inhaler technique intervention by community pharmacists. *Journal of Allergy and Clinical Immunology*.

[96] Burton AJ (1984). Asthma inhalation devices: what do we know?. *British Medical Journal*.

[97] Interiano B, Guntupalli KK (1993). Metered-dose inhalers: do health care providers know what to teach?. *Archives of Internal Medicine*.

[98] Broeders MEAC, Sanchis J, Levy ML, Crompton GK, Dekhuijzen PNR (2009). The ADMIT series—issues in inhalation therapy. 2) Improving technique and clinical effectiveness. *Primary Care Respiratory Journal*.

[99] Kim S-H, Kwak HJ, Kim T-B (2009). Inappropriate techniques used by internal medicine residents with three kinds of inhalers (a metered dose inhaler, diskus, and turbuhaler): changes after a single teaching session. *Journal of Asthma*.

[100] Basheti IA, Armour CL, Reddel HK, Bosnic-Anticevich SZ (2009). Long-term maintenance of pharmacists’ inhaler technique demonstration skills. *American Journal of Pharmaceutical Education*.

[101] Duerden M, Price D (2001). Training issues in the use of inhalers. *Disease Management and Health Outcomes*.

[102] van der Palen J, Klein JJ, Kerkhoff AH, van Herwaarden CL, Seydel ER (1997). Evaluation of the long-term effectiveness of three instruction modes of inhaling medicines. *Patient Education and Counseling*.

[103] De Blaquiere P, Christensen DB, Carter WB, Martin TR (1989). Use and misuse of metered-dose inhalers by patients with chronic lung disease: a controlled, randomized trial of two instruction methods. *American Review of Respiratory Disease*.

[104] Nimmo CJR, Chen DNM, Martinusen SM (1993). Assessment of patient acceptance and inhalation technique of a pressurized aerosol inhaler and two breath-actuated devices. *Annals of Pharmacotherapy*.

[105] Hardwell A, Barber V, Hargadon T, McKnight E, Holmes J, Levy ML (2011). Technique training does not improve the ability of most patients to use pressurised metered-dose inhalers (pMDIs). *Primary Care Respiratory Journal*.

[106] Takemura M, Kobayashi M, Kimura K (2010). Repeated instruction on inhalation technique improves adherence to the therapeutic regimen in asthma. *Journal of Asthma*.

[107] Gray SL, Williams DM, Pulliam CC, Sirgo MA, Bishop AL, Donohue JF (1996). Characteristics predicting incorrect metered-dose inhaler technique in older subjects. *Archives of Internal Medicine*.

[108] Agertoft L, Pedersen S (1998). Importance of training for correct Turbuhaler use in preschool children. *Acta Paediatrica, International Journal of Paediatrics*.

[109] van der Palen J, Klein JJ, van Herwaarden CLA, Zielhuis GA, Seydel ER (1999). Multiple inhalers confuse asthma patients. *European Respiratory Journal*.

[110] Molimard M, Gros VL (2008). Impact of patient-related factors on asthma control. *Journal of Asthma*.

[111] Al-Showair RAM, Pearson SB, Chrystyn H (2007). The potential of a 2Tone trainer to help patients use their metered-dose inhalers. *Chest*.

[112] Lavorini F, Levy ML, Corrigan C, Crompton G (2010). The ADMIT series-issues in inhalation therapy. 6) training tools for inhalation devices. *Primary Care Respiratory Journal*.

[113] Thomas M, Price D, Chrystyn H, Lloyd A, Williams AE, von Ziegenweidt J (2009). Inhaled corticosteroids for asthma: impact of practice level device switching on asthma control. *BMC Pulmonary Medicine*.

